# Sustained feeding of a diet high in fat resulted in a decline in the liver's insulin-degrading enzyme levels in association with the induction of oxidative and endoplasmic reticulum stress in adult male rats: Evaluation of 4-phenylbutyric acid

**DOI:** 10.1016/j.heliyon.2024.e32804

**Published:** 2024-06-10

**Authors:** Fateme Binayi, Behnam Saeidi, Fatemeh Farahani, Mina Sadat Izadi, Farzaneh Eskandari, Fariba Azarkish, Mohammad Sahraei, Rasoul Ghasemi, Fariba Khodagholi, Homeira Zardooz

**Affiliations:** a Neurophysiology Research Center, Shahid Beheshti University of Medical Sciences, Tehran, Iran; b Department of Physiology, School of Medicine, Shahid Beheshti University of Medical Sciences, Tehran, Iran; c Protein Research Center, Shahid Beheshti University, Tehran, Iran; d Department of Physiology, Faculty of Medical Sciences, Tarbiat Modares University, Tehran, Iran; e Department of Physiology, School of Medicine, Isfahan University of Medical Sciences, Isfahan, Iran; f School of Dentistry, Shahid Beheshti University of Medical Sciences, Tehran, Iran; g Neuroscience Research Center, Shahid Beheshti University of Medical Sciences, Tehran, Iran

**Keywords:** High fat diet, Endoplasmic reticulum and oxidative stress, Insulin degrading enzyme, 4-Phenylbutyric acid

## Abstract

The current study explored the impact of high fat diet (HFD) on hepatic oxidative and endoplasmic reticulum (ER) stress and its insulin degrading enzyme (IDE) content with the injection of 4-phenyl butyric acid (4-PBA) in adult male rats.

Following the weaning period, male offspring were distributed among six distinct groups. The corresponding diet was used for 20 weeks, subsequently 4-PBA was administered for three consecutive days. Plasma glucose and insulin levels, HOMA-β (homeostasis model assessment of β-cell), hepatic ER and oxidative stress biomarkers and IDE protein content were assessed.

Long-term ingestion of HFD (31 % cow butter) induced oxidative and ER stress in the liver tissue. Accordingly, a rise in the malondialdehyde (MDA) content and catalase enzyme activity and a decrease in the glutathione (GSH) content were detected within the liver of the HFD and HFD + DMSO groups. Consumption of this diet elevated the liver expression of binding immunoglobulin protein (BIP) and C/enhancer-binding protein homologous protein (CHOP) levels while reduced its IDE content. The HOMA-β decreased significantly. The injection of the 4-PBA moderated all the induced changes.

Findings from this study indicated that prolonged HFD consumption led to a reduction in plasma insulin levels, likely attributed to pancreatic β cell malfunction, as evidenced by a decline in the HOMA-β index. Also, the HFD appears to have triggered oxidative and ER stress in the liver, along with a decrease in its IDE content.

## 1 Introduction

Prolonged feeding of high fat diet (HFD) triggers endoplasmic reticulum (ER) and oxidative stress in the liver. There are also some reports based on the involvement of ER and oxidative stress in the occurrence of metabolic disorders like obesity and diabetes [[1], [2], [3]]. The liver as one of the primary locations responsible for removing insulin from the body play a crucial part in regulating the systemic insulin concentrations [4,5]. Insulin clearance in the liver is mostly done through the activity of insulin-degrading enzyme (IDE), that breaks down nearly 50 % of circulating insulin during the primary passage through the hepatic portal system [6]. IDE enzyme is a zinc-dependent metalloproteinase positioned within cytosol, peroxisomes, endosomes, and cell surface. This enzyme is responsible for breaking down insulin and amyloidogenic peptides, and it plays multiple roles in maintaining glucose and insulin homeostasis, including the control of insulin release from pancreatic β-cells. Additionally, several studies have shown that IDE influences the action and sensitivity of insulin in the liver [7]. In this regard, Venturini and colleagues has demonstrated that elevated IDE mRNA level in the liver of rat was linked to ameliorated glucose tolerance and insulin sensitivity [8]. In contrast, reduced expression of IDE is linked to the incidence of type 2 diabetes along with hyperinsulinemia [9]. This finding aligns with the observation that the suppression of insulin signaling triggers a decrease in IDE expression [10]. Increasing the activity and mRNA expression of the IDE enzyme [11], and conversely, attenuation of the IDE gene and protein expressions [12] following cafeteria diet consumption have been reported. IDE enzyme is sensitive to the redox status of the cell, so that enhancement of reactive oxygen species (ROS) directly and also through the increase of oxidized glutathione (Glutathione disulfide: GSSG) decreased the IDE activity, while the increase of reduced glutathione (GSH) had the opposite effect and augmented the breakdown of insulin [6]. In this context, the use of Nigella sativa oil with antioxidant properties in rats suffering from type 2 diabetes brought the content of oxidant and antioxidant factors close to the control value and probably through the improvement of the pathway of insulin signaling increased IDE amount and reduced amyloidogenic markers in the brains of these animals [13]. Furthermore, the (use of taurine conjugated bile acid, tauroursodeoxycholic (TUDCA), which prevents the occurrence of oxidative stress and reduces ER stress in C57Bl/6 mice that were obese and hyperinsulinemic with a HFD, improved insulin signaling and increased protein levels of IDE enzyme followed by enhanced insulin clearance [14]. Conversely, some investigations have presented the 4-phenylbutyric acid (4-PBA) as a chemical chaperone, which is safe for administrating *in vivo*, can inhibit pathways of endoplasmic reticulum (ER) and oxidative stress (two paths that interact together) and also improve the disturbance in insulin secretion caused by consuming HFD [[15], [16], [17]].

Chronic consumption of high-fat food, possibly by inducing hepatic oxidative and ER stress, can reduce the amount of IDE enzyme protein and hepatic clearance of insulin and eventually result in hyperinsulinemia. On the other hand, considering that the 4-PBA drug has inhibitory effects on oxidative and ER stress, it can probably have a modulating effect on the liver content of the IDE enzyme and correct the plasma insulin content. Since limited investigations have evaluated the above hypotheses and conflicting findings have been observed, we explored the impact of oxidative and ER stress as a result of chronic ingestion of HFD (comprising 31 % of cow butter by weight) on the content of hepatic IDE in adult male rats with and without of 4-PBA treatment. For this purpose, the liver oxidative stress factors including malondialdehyde (MDA) and GSH contents, and ER stress indicators such as C/enhancer-binding protein homologous protein (CHOP) and binding immunoglobulin protein (BIP), as well as catalase enzyme activity and IDE content were measured. Furthermore, plasma levels of glucose and insulin in fasting status were measured and the homeostasis model assessment of β-cell (HOMA-β) biomarker was calculated as a marker of the pancreatic beta cell's secretory function.

## 2 Materials and methods

### 2.1 Animals

Female (200 ± 30 g) and male (250 ± 50 g) Wistar rats (Pasteur Institute, Iran) were employed in this investigation. Rats were kept under 12h light/dark cycle, stable temperature and humidity (23 ± 2 °C and 65 ± 5 %, respectively), and unrestricted availability of food and water. The current work was done in accordance with the “Ethics Committee of Shahid Beheshti University of Medical Sciences” (IR.SBMU.PHNS.REC.1401.134) which is in compliance with the standards related to working with laboratory animals.

The rats were housed together in a single cage for mating, consisting of one male and two females. After confirming pregnancy (by preparing a vaginal smear and observing sperm), the male rat was taken out of the cage, and the female rat was left alone until delivery in an individual cage. After giving birth, the female rats were cared for until the end of the lactation period, observing the number of 6–8 offspring per birth. After weaning the male offspring (n = 36 rats) were allocated randomly into six groups (n = 6rats/group) regarding the diet type [normal diet (ND) or HFD] and receiving drug (4-phenylbutyric acid: 4-PBA or its solvent dimethyl sulfoxide: DMSO). The rats' body weight and food consumption were recorded biweekly from postnatal day 22 until the end of the experiment with a digital weighing device (Amput, China, with a 1-g resolution). The food consumption was determined by subtracting the remaining food from the complete amount of food allocated in the cage after 24 h.

### 2.2 Experimental groups

The six study groups are as follows.

1 ND: The rats that consumed a ND from the end of weaning to the end of experimental period.
2 ND + DMSO: The rats maintained on a ND for 20 weeks, beginning after weaning and lasting until the start of the 21st week, were injected with DMSO two times daily (three successive days).
3 ND+4-PBA: The rats maintained on a ND for 20 weeks, beginning after weaning and lasting until the start of the 21st week, were injected with 4-PBA two times daily (three successive days).
4 HFD: The rats that consumed a HFD for 20 weeks, beginning after weaning and lasting until the start of the 21st week.
5 HFD + DMSO: The rats maintained on a HFD for 20 weeks, beginning after weaning and lasting until the start of the 21st week, were injected with DMSO two times daily (three successive days).
6 HFD+4-PBA: The rats maintained on a HFD for 20 weeks, beginning after weaning and lasting until the start of the 21st week, were injected with 4-PBA two times daily (three successive days).

### 2.3 Preparation of high-fat diet

To prepare high fat food, after grinding the standard pellets (Behparvar animal feed producing Company, Iran), butter from cow (31 % by weight), soy protein (4 %), and vitamins and minerals (0.7 %) were added. Then the final mixture was ground and given to the animals in the form of pellets [17].

The DMSO and 4-PBA groups received DMSO (1.02952.1000, Merck, Germany) or a 50 mg/kg dose of 4-PBA (P21005, Sigma Aldrich, Germany) via IP injection at the start of the 21st week. Both groups were injected twice daily at 10:00 and 13:00 for three consecutive days [18].

### 2.4 Blood sampling and measurement of plasma parameters

All the rats (6rats/group) were lightly anesthetized using isoflurane (Baxter, USA), in fasting conditions (14–16 h), then blood sampling was done by cutting the end of tail at 8:00–8:30 [17,19]. Blood was gathered into 1.5 mL microtubes containing a heparin solution of 10 μL/mL (5000 IU/mL) [20], and centrifuged at 664×*g* for 10 min. Following this, the plasma was extracted and kept frozen at −70 °C for glucose and insulin level determination [21].

#### 2.4.1 Measurement of glucose, insulin, and the HOMA-β markers

Plasma glucose (6rats/group) was measured through the glucose oxidase procedure with 5 mg/dL sensitivity (Pars Azmoon, Iran). Rat insulin ELISA Kit (sensitivity 0.07 μg/L, Mercodia, Sweden) was utilized to determine plasma insulin concentration.

The HOMA-β indices was determined by the below formula [22]:

HOMA−β%=[20×fastinginsulin(μU/mL)/fastingglucose(mM)−3.5]×100

### 2.5 Preparation of liver tissue and measurement of protein factors

One day after the last 4-PBA injection, the animals (6rats/group), following 12–14h fasting, were put under anesthesia with isoflurane and subsequently euthanized by decapitation. The trunk blood was then allowed to drain, and their liver and intra-abdominal fat were rapidly extracted, rinsed with normal saline, and their weights recorded.

Rats’ liver tissue homogenized using a laboratory homogenizer (TOMY Micro Smash MS 100, Indonesia) in the lysis buffer. The resulting solution underwent centrifugation at 12000 rpm for 30 min at 4 °C. Then, the supernatant was gathered and stored at −70 °C until the assessment of oxidative stress and ER stress markers [9]. The Bradford procedure was utilized to measure protein content in the supernatant [23,24].

#### 2.5.1 Measurement of MDA, GSH and catalase activity of liver tissue

The hepatic MDA level, as an oxidative stress marker, was measured by the ELISA method using an MDA commercial colorimetric kit with 0.1 μM sensitivity (Zellbio, Germany). The MDA value was reported in mg of protein [25].

Ellman method was applied to determine the amount of GSH of liver tissue. In this method, the volume of the solution resulted from the homogenized tissue (containing 120 μg protein) was brought to 200 μL through the addition of a phosphate buffered-saline (PBS). Trichloroacetic acid subsequently was added and the mixture was centrifuged. In the next step, 0.1 ml DTNB [5, 5ˋ-Dithiobis-2-Nitrobenzoic acid (2 mg/mL PBS)] (D218200, Sigma, USA) was added to the prepared supernatant and allowed to incubated at 37 °C for 10 min. Finally, the light absorption was quantified by an ELISA reader (BioTeK, ELX800TS, USA) at a wavelength of 405 nm [26,27]. GSH concentration was presented as μmol per mg of protein. Catalase activity in the liver tissue was measured employing the Goth technique. In short, PBS buffer was mixed with the supernatant encompassing 180 μg of protein. Afterward, hydrogen peroxide (H_2_O_2_, 0.01 M) was added to this mixture and incubated for 15 min at 25 °C in the dark. Then, ammonium molybdate (6.35 mg/mL PBS) (277908, Sigma, USA) was introduced to halt the reaction, and the light absorption was read using an ELISA reader at 405 nm wavelength. The findings were reported as μmol H_2_O_2_/min per mg protein [[28], [29], [30]].

#### 2.5.2 Measurement of ER stress biomarkers (BIP and CHOP) in the liver tissue

In order to assess the BIP and CHOP biomarkers levels, the Western blot technique was applied. First, liver samples (4rats/group) were lysed using a lysis buffer comprising Tric-HCl pH = 8 sodium deoxycholate, NaCl, sodium dodecyl sulfate (SDS), ethylenediamine tetra-acetic acid (EDTA), Triton and H_2_O. The resulting lysate was subsequently centrifuged for 30 min at 12000 rpm to eliminate cellular debris. Then the supernatant was collected and the Bradford protocol was utilized to measure the total protein content [31]. The specimens (comprising 60 μg of protein) on the SDS-PAGE (sodium dodecyl sulfate-polyacrylamide gel electrophoresis, 12%Bis–Tris Plus gels) were electrophoresed (n = 4rats/group). In the next step, the isolated proteins were moved from the gel onto a Polyvinylidene Fluoride (PVDF) paper based on molecular weight using the semi-wet method. After that using primary antibodies of BIP (ab227865, Abcam, UK) and CHOP (M00311, Abcam, UK) (1 mg/mL) the expression of BIP and CHOP proteins levels were measured. A β-actin antibody (ab8227, Abcam, UK) was used as a loading control. The secondary anti-rabbit IgG antibody (CS7074, Cell Signaling, USA) was diluted to a concentration ratio of 1:1500. In the end, the protein bands were detected using an electrochemiluminescence kit (Amersham ECL select™, GE Healthcare, USA) [17].

#### 2.5.3 Measurement of IDE enzyme content in the hepatic tissue

The hepatic tissue was prepared for IDE enzyme protein measurement according to the method outlined in section 2.5. The resulting supernatant from the liver tissue (6rats/group) was utilized to measure the total protein with the Bradford procedure and the IDE enzyme protein content with an ELISA kit using a colorimetric assay (Zellbio, Germany). The enzyme was reported as nanograms per milligram of protein.

### 2.6 Data analysis methods

All data was analyzed by GraphPad Prism software, version 8.0 (GraphPad Software, USA). Data distribution was assessed using Kolmogorov–Smirnov test; it was normal in all study groups. Results were demonstrated as Mean ± standard error of mean (SEM) and p < 0.05 was regarded as statistically meaningful. Two-way ANOVA followed by Tukey's post hoc test was utilized to differentiate between distinct groups (considering the independent factors: diet and drug). To compare the alterations in body weight and food consumption over time between different groups, a repeated measures ANOVA (considering diet and drug as independent factors and time as repeated factor) was used. Also, area under the curve (AUC) of these parameters was calculated through Excel software in each group, and the statistical test of two-way ANOVA followed by Tukey's post hoc test was done.

## 3 Results

### 3.1 The impact of HFD and/or 4-PBA on body weight and food consumption

**Fig. 1:**
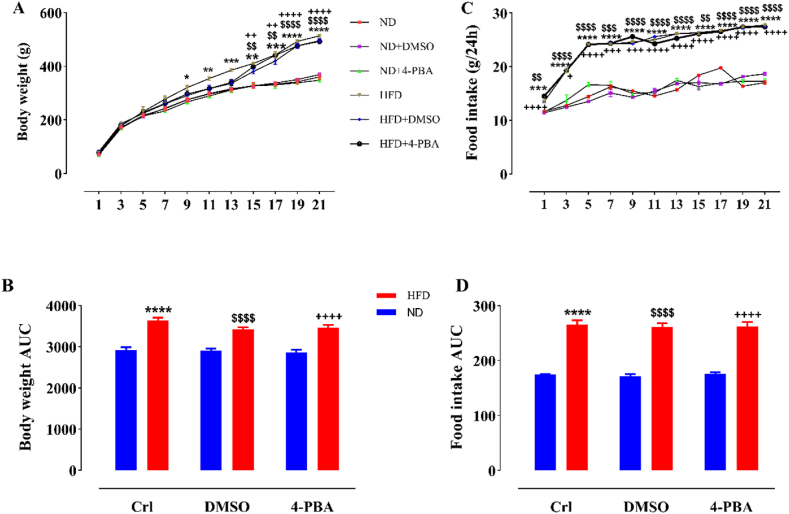
The impact of high fat diet and/or 4-PBA on body weight changes (A), body weight AUC (B), amount of food intake alterations (C), and food intake AUC (D) during the experimental period. The column/point demonstrates Mean ± SEM for 6 animals. ****P < 0.0001, ***P < 0.001, **P < 0.01, *P < 0.05 substantial distinction with control group consuming normal diet, ^$$$$^P < 0.0001, ^$$$^P < 0.001, ^$$^P < 0.01 substantial distinction with ND + DMSO group, ^++++^ P<0.000, ^+++^ P<0.001, ^++^ P<0.01,+P<0.05 substantial distinction with ND+4-PBA group. Crl: Control, ND: Normal Diet, HFD: High-Fat Diet, 4-PBA: 4-Phenyl Butyric Acid and DMSO: Dimethyl Sulfoxide.

As shown in Fig. 1 (A), HFD consumption resulted in a substantial elevation of body weight in HFD groups, from week 9–21 of the experimental period, compared to ND groups. Also, the area under the curve (AUC) of body weight alteration alterations in the HFD-consuming groups relative to the normal diet (ND) groups increased markedly (P < 0.0001, Fig. 1B). The injection of 4-PBA and its solvent DMSO did not cause a remarkable change in body weight in the HFD- and ND-consuming rats compared with the controls (Fig. 1A and B).

Statistical analysis revealed that consumption of HFD resulted in a significant enhancement of food intake in HFD groups, from week 1–21 of the experimental period, in relation to the ND groups (Fig. 1C). Moreover, food intake AUC increased considerably (P < 0.0001, Fig. 1 D) following HFD feeding. 4-PBA and DMSO injection did not affect the food intake in ND- and HFD groups relative to the control groups (i.e., ND and HFD groups, respectively) (Fig. 1C and D).

### 3.2 The impact of HFD and/or 4-PBA on intra-abdominal fat and liver weight

**Fig. 2:**
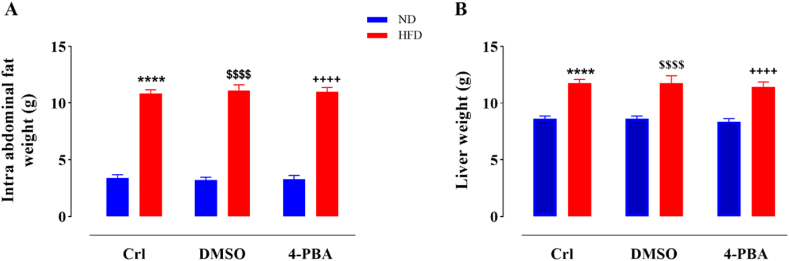
Impact of high-fat diet and/or 4-PBA on intra-abdominal fat (A) and liver (B) weight. The column demonstrates Mean ± SEM for 6 animals. ****P < 0.0001 substantial distinction with control group consuming normal diet, ^$$$$^P < 0.0001 substantial distinction with ND + DMSO group, and ^++++^P<0.0001 substantial distinction with ND+4-PBA group. Crl: Control, ND: Normal Diet, HFD: High-Fat Diet, 4-PBA: 4-Phenyl Butyric Acid and DMSO: Dimethyl Sulfoxide.

HFD consumption markedly elevated the intra-abdominal fat mass and liver weight in the HFD-received animals relative to the normal diet group (p < 0.0001) (Fig. 2A and B). DMSO- and 4-PBA-treated rats in the ND-fed and HFD-fed groups did not show a remarkable change in the of intra-abdominal fat mass and liver weight in contrast to the ND and HFD control groups, respectively (Fig. 2A and B).

### 3.3 The impact of HFD and/or 4-PBA on plasma glucose and insulin concentrations, in fasting status, and HOMA-β index

**Fig. 3:**
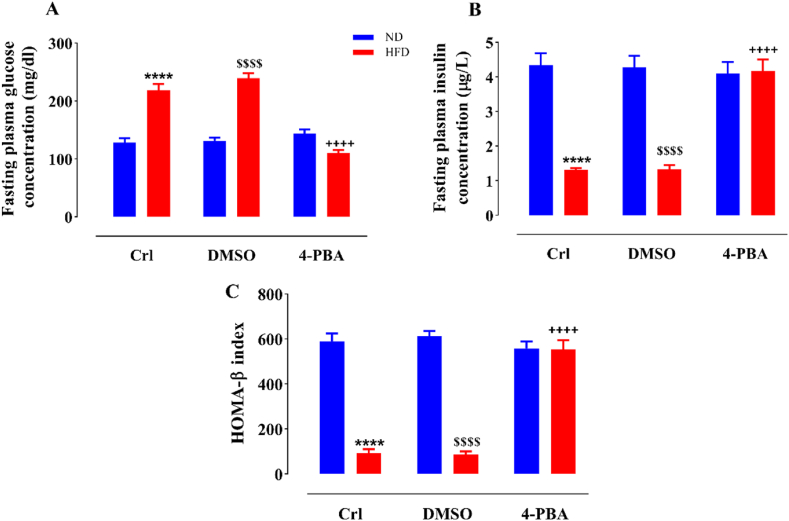
The impact of HFD and/or 4-PBA on fasting plasma concentrations of glucose (A), insulin (B) and HOMA-β index (C). The column demonstrates Mean ± SEM for 6 animals. ****P < 0.0001 substantial distinction with the normal diet consuming group, ^$$$$^P < 0.0001 substantial distinction with the ND + DMSO group, and ^++++^P<0.0001 substantial distinction with HFD and HFD + DMSO groups. Crl: Control, ND: Normal Diet, HFD: High-Fat Diet, 4-PBA: 4-Phenyl Butyric Acid, DMSO: Dimethyl Sulfoxide and HOMA-β: Homeostasis Model Assessment of β-cell.

Consuming a HFD caused a significant augmentation in fasting plasma glucose concentration (P < 0.0001) and a substantial decline in fasting plasma insulin level (P < 0.0001), in the HFD and HFD + DMSO groups in comparison to the ND and ND + DMSO groups, respectively (Fig. 3A and B). The 4-PBA administration in the HFD+4-PBA group resulted in a significant reduction in the fasting plasma glucose concentration and a significant increase in the fasting plasma insulin level in comparison to the HFD and HFD + DMSO groups, such that it did not exhibit any notable difference with all of the ND groups (P < 0.0001) (Fig. 3A and B). The administration of DMSO did not induce a notable alteration in glucose and insulin plasma levels relative to their respective controls in any of the dietary groups (Fig. 3A and B).

HFD intake led to a notable decrease in HOMA-β in the HFD and HFD + DMSO rats relative to the ND and ND + DMSO ones (P < 0.0001) (Fig. 3C). The injection of 4-PBA in the HFD+4-PBA group led to a marked rise in HOMA-β index (P < 0.0001) compared with the HFD and HFD + DMSO groups, while it did not exhibit any significant difference in comparison to all the rats that consumed ND (Fig. 3C). On the other hand, administration of DMSO did not cause a significant change in HOMA-β index in none of the diets in relation to the control groups (ND + DMSO and HD + DMSO groups vs. ND and HD groups, respectively) (Fig. 3C).

### 3.4 The impact of HFD and/or 4-PBA on liver MDA and GSH levels and catalase activity

**Fig. 4:**
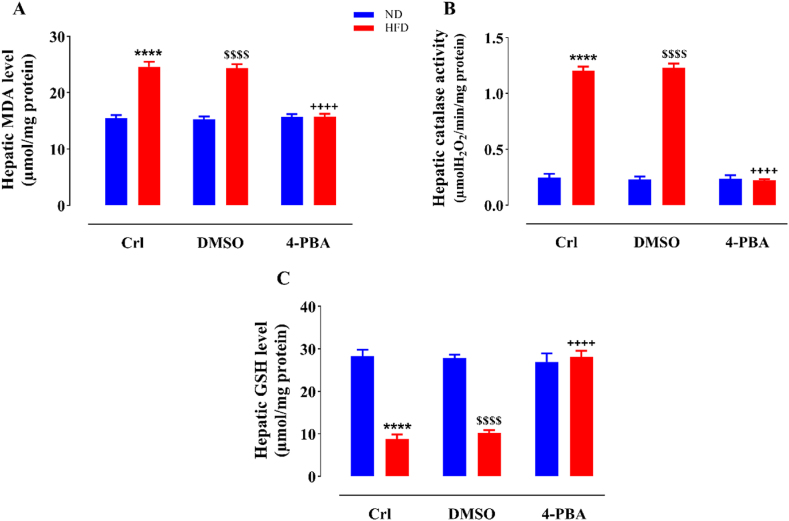
The impact of HFD and/or 4-PBA on the content of liver MDA level (A), catalase activity (B) and GSH level (C) . The column demonstrates Mean ± SEM for 6 animals. ****P < 0.0001 substantial distinction with the control group consuming normal diet, ^$$$$^P < 0.0001 substantial distinction with the ND + DMSO group, and ^++++^P<0.0001 substantial distinction with HFD and HFD + DMSO groups. Crl: Control, ND: Normal Diet, HFD: High-Fat Diet, 4-PBA: 4-Phenyl Butyric Acid, DMSO: Dimethyl Sulfoxide, MDA: Malondialdehyde and GSH: reduced Glutathione.

Consuming a HFD substantially elevated MDA levels (Fig. 4A) and catalase enzyme activity (Fig. 4B) in the hepatic tissue of HFD and HFD + DMSO rats relative to ND and ND + DMSO ones, respectively (P < 0.0001). The administration of 4-PBA in the HFD+4-PBA group caused a marked decline in these variables relative to the HFD and HFD + DMSO groups, resulting in no significant difference when compared to the animals fed a normal diet (P < 0.0001) (Fig. 4A and B). DMSO administration did not cause a notable alteration in MDA content and catalase enzyme activity relative to the control groups in none of the diets (Fig. 4A and B). With the consumption of HFD, GSH content in the liver tissue of HFD and HFD + DMSO groups diminished profoundly in comparison to the ND and ND + DMSO groups (P < 0.0001) (Fig. 4C). The administration of 4-PBA in the HFD+4-PBA group led to a notable rise in this parameter relative to the HFD and HFD + DMSO groups (P < 0.0001) such that in contrast with the ND-fed rats did not exhibit any significant distinction. The injection of DMSO did not lead to a significant alteration in GSH content in comparison to the control groups in any of the dietary groups (i.e. ND + DMSO and HD + DMSO groups vs. ND and HD groups, respectively) (Fig. 4C).

### 3.5 The impact of HFD and/or 4-PBA on ER stress biomarkers

**Fig. 5:**
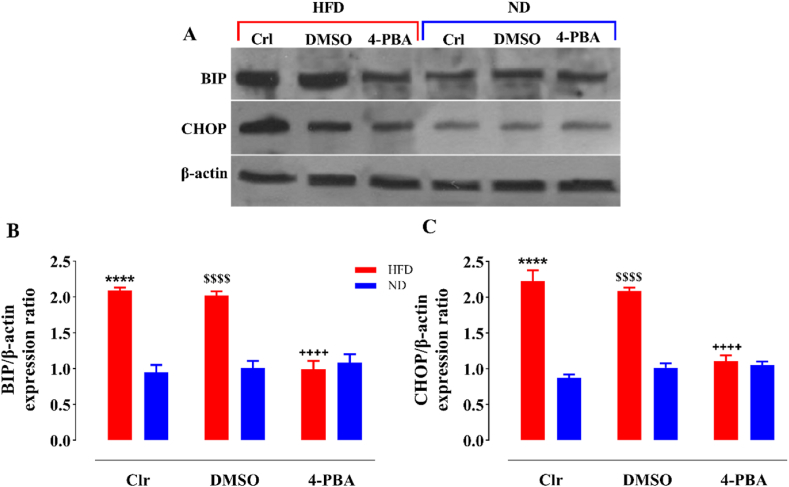
The impact of HFD and/or 4-PBA on BIP (A, B) and CHOP (A, C) protein expression in the liver tissue. The column demonstrates Mean ± SEM for 4 animals. ****P < 0.0001 substantial distinction with the control group consuming a normal diet, ^$$$$^P < 0.0001 substantial distinction with the ND + DMSO group, and ^++++^P<0.0001 substantial distinction with HFD and HFD + DMSO groups. The original non-adjusted images of Western blot are presented as supplementary file named “full images of Western blot”. Crl: Control, ND: Normal Diet, HFD: High-Fat Diet, 4-PBA: 4-Phenyl Butyric Acid, DMSO: Dimethyl Sulfoxide, BIP: Binding Immunoglobulin Protein, CHOP: C/enhancer-binding protein Homologous Protein.

According to our finding, HFD led to a notable rise in the expression of BIP (Fig. 5A and B) and CHOP (Fig. 5A–C) proteins in the liver tissue of the HFD and HFD + DMSO groups compared to the ND and ND + DMSO groups (P < 0.0001). The injection of 4-PBA attenuated the expression of BIP (P < 0.0001) and CHOP (P < 0.0001) proteins in this tissue compared with the HFD and HFD + DMSO groups, while there was no notable distinction with the ND-fed rats. Also, DMSO-injected rats did not show remarkable differences in the amount of BIP and CHOP proteins related to the control groups in any of the diets (Fig. 5).

### 3.6 The impact of HFD and/or 4-PBA on liver IDE level

**Fig. 6:**
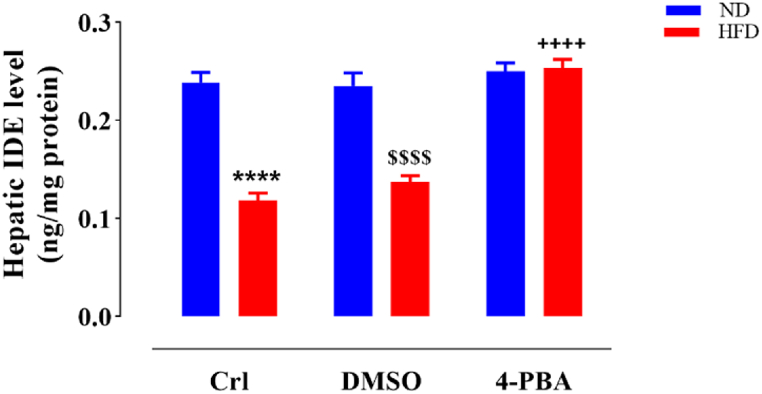
The impact of HFD and/or 4-PBA on liver IDE content. The column demonstrates Mean ± SEM for 6 animals. ****P < 0.0001 substantial distinction with the normal diet received rats, ^$$$$^P < 0.0001 substantial distinction with the ND + DMSO group, and ^++++^P<0.0001 substantial distinction with HFD and HFD + DMSO groups. Crl: Control, ND: Normal Diet, HFD: High-Fat Diet, 4-PBA: 4-Phenyl Butyric Acid, DMSO: Dimethyl Sulfoxide and IDE: Insulin-Degrading Enzyme.

The IDE content in the liver tissue was significantly lower in rats fed a HFD and HFD + DMSO related to those on a ND and ND + DMSO (P < 0.0001) (Fig. 6). Injecting 4-PBA into the HFD+4-PBA group led to a marked elevation of IDE content relative to the HFD and HFD + DMSO groups (P < 0.0001), such that compared to the normal diet received rats did not show any significant difference (Fig. 6). The injection of DMSO did not notably altered IDE content in comparison to the control groups in any of the diets (ND + DMSO and HFD + DMSO) (Fig. 6).

## 4 Discussion

In this investigation, chronic intake of a HFD, comprising 31 % cow butter, induced ER and oxidative stress in the male adult rat's liver tissue. According to this finding, MDA content increased and GSH content decreased along with the enhancement of catalase enzyme activity in the liver tissue. Also, the protein expression of the BIP and CHOP biomarkers was increased and hepatic IDE content showed a significant attenuation. These variations were associated with an increase in body weight, amount of food intake, intra-abdominal fat weight, and liver weight. Moreover, the plasma glucose level increased, however the plasma insulin level and HOMA-β index were remarkably decreased and 4-PBA treatment moderated all these changes and brought the values closer to the control value.

In agreement with the current study, findings of an investigation in which male rats (initial weight: 250–300 g) had free access to a HFD for 11 weeks, demonstrated an increase in body weight and lipid droplets in liver tissue cells (steatosis), as well as oxidative stress in this tissue. In this regard, enhancement of the MDA content and carbonylated protein level along with reduction of superoxide dismutase (SOD), catalase and glutathione peroxidase activity as the oxidative stress-related alterations, were found in the liver of these animals [32]. Additionally, HFD intake for two months in the male Wistar rats resulted in elevated body weight and plasma levels of insulin and glucose, accompanied by augmentation of lipids peroxidation and carbonylated protein level in plasma. In these animals, an increase in ROS production and oxidative stress was revealed in liver and pancreas tissues [33]. On the other hand, following a HFD consumption for 30 days body weight, energy intake, plasma glucose, dyslipidemia, and oxidized-LDL in the liver tissue were increased while no significant change was reported in GSH content and activity of SOD and catalase enzymes [34]. Different results of various studies can be caused by the difference in the start time or duration of the HFD intake. It is essential to note that HFD consumption can lead to the accumulation of fatty acids like palmitate and lipotoxicity and the enhancement of toxic types such as ceramides and diacylglycerol in the hepatocytes. Studies have reported that ceramides increment leads to mitochondrial dysfunction; inhibition of beta-oxidation of fatty acids and elevation of the ROS production in the mitochondria, which in turn disrupts the balance of antioxidants in liver cells. Redox system imbalance affects the antioxidant response and leads to reduction of the antioxidant levels (such as GSH and SOD) [35]. In addition, increase in fatty acid levels in hepatocytes induces triggers ER stress and increases unfolded protein response (UPR) signaling which in turn, results in enhanced ROS production and lipid peroxidation. As far as, ER serves a crucial role in regulating lipid metabolism in the liver, disruption of ER homeostasis can activate lipogenic enzymes, induce hepatic steatosis, and intensify oxidative stress [32]. Moreover, HFD-induced hyperglycemia increases the enzymatic activity of glucose-6-phosphatase (G6Pase) in the liver cells. Following the increase of glucose-6-phosphate, as a glucose metabolite, the transcription factors involved in lipogenesis are activated which leads to lipogenesis, fat accumulation, lipid metabolism disorder, and finally the incidence of oxidative damage in the hepatic tissue [36]. In this respect, in human liver cells (HepG2) which were located in a culture medium containing 20 mM d-glucose for 24h and then exposed to 100 nM insulin for 10 min, ROS and carbonylated protein levels increased and the GSH content decreased. At higher doses of glucose (30–60 mM), catalase and glutathione peroxidase activities increased. In fact, high concentrations of glucose caused an excessive production of ROS in these cells which directly lead to oxidative damage to proteins and the reduction of GSH content. Augmented antioxidant activity is also an adaptive response to counteract excessive ROS production and oxidative stress resulted from hyperglycemia [37].

Chronic consumption of a HFD in most studies caused a noteworthy increase in the quantity of food consumed and body weight in HFD-received groups [[38], [39], [40], [41]], which is in agreement with our results. The body weight enhancement following the consumption of a HFD is possibly due to the increase of fat accumulation in the body and its organs. In this respect, the present study showed an enhancement of intra-abdominal fat and liver weight in the HFD-consuming groups. Previous investigations have demonstrated that HFD consumption can result in higher lipid droplet as lipid storage in the liver, which is typically linked to the liver and body weight elevation [42,43]**.** In our study we did not assess liver lipid droplets as lipid storage; therefore, we recommend that these parameters be measured in future studies. Contrary to these findings, it has been reported that consumption of 20 percent corn oil for 12 weeks decreased food intake in adult female rats, although the body weight of the animals had increased [40]. In another study, HFD-intake containing 58 % unsaturated fat for seven weeks reduced food intake and body weight in three-week-old C57Bl/6J male mice [44]. The discrepancies in the findings of variance investigations could be related to differences in the kind of fat, lengths of fat consumption, and gender. Administration of 4-PBA could not significantly affect the body weight and food consumption. However, some reports have supported that 4-PBA increases the activity of brown fat tissue and consequently energy consumption and reduces body weight. Different effects of 4-PBA on body weight in different studies can be due to the discrepancy in the dosage of the drug (500 mg/kg/day) [45,46] or the duration of drug usage including 8 weeks [45] and 14 days [46]. In the present study, 4-PBA was injected in a shorter time (3 days) and a lower dosage (50 mg/kg/day), and probably because of this, it could not change the body weight and amount of food consumption in HFD-fed rats.

Our findings revealed that HFD consumption resulted in the enhancement of glucose plasma level and reduction of insulin plasma level in fasting state and HOMA-β index. Consistent with these results, previous studies have shown that intake of HFD comprising 40 % lard fat in 6-week-old Wistar rats for 8 weeks [47,48] and in 4-week-old ones for 21 weeks [49,50] caused the increment of blood glucose, sympathetic nervous system activity, and the level of norepinephrine in the pancreas, liver, and brain, while decreased the level of blood insulin and glucose absorption in adipose tissue and skeletal muscles. On the other hand, in another investigation consumption of HFD comprising 60 % lard fat for 8 weeks elevated plasma glucose and did not alter insulin in rat [51]. Another study on adult male Wistar rats revealed that intake of HFD comprising 24 % lard fat for 4 weeks increased fasting plasma insulin concentration, showed no alteration fasting plasma glucose level, and impaired glucose and insulin tolerance [52]. It is important to mention that some studies have indicated that feeding mice with HFD containing 60 % lard for 3, 6, or 12 months can cause insulin resistance and increased plasma insulin levels [53,54]. In light of this, a 60 % lard diet may be a more effective approach for examining the impact of 4-PBA on IDE in the context of insulin resistance. The reason for the difference in these results can be the different types, percentages, and/or length of fat intake. Based on the aforementioned investigations, a rise in sympathetic tone and the resulting elevated release of norepinephrine originating from sympathetic nerve endings in pancreatic tissue could be one of the contributing factors in reducing plasma insulin levels observed in animals consuming HFD. Norepinephrine released under these conditions has been shown to suppress insulin release from pancreatic β cells [47,48,55]. Additionally, it was demonstrated that being exposed to saturated fatty acids like palmitate, which also accounts for a significant portion (38.3 %) of the fat utilized in this study leads to the activation of UPR proteins and the occurrence of ER stress [[56], [57], [58], [59]]. In this regard, Choi et al. demonstrated that the culture of INS-1 cells from rats that were fed with palmitate prompted ER stress and inhibited GSIS (glucose-stimulated insulin secretion) in these cells [60]. Induced oxidative and ER stress in β cells result in the dysfunction of these cells and consequently a decrease in secretion of insulin in response to basal and high levels of glucose in the HFD-consuming groups [61,62]. According to the reasons mentioned above, in the present investigation, the decrease in insulin plasma level is probably due to the induction of ER and oxidative stress in the pancreas tissue of HFD-consuming rats, which was shown in our previous research [17]. In this respect, the results of HOMA-β also exhibited the dysfunction of beta cells in the HFD-consuming groups. Consistent with this finding, some investigations reported a decrease in the HOMA-β index as a consequence of HFD intake, including HFD containing 57 % fat for 20 weeks in adult male mice [63], HFD containing 44.6 % lard fat for 8 weeks [64] and HFD containing 53 % sheep fat for 28 days in male Wistar rats [65]. In contrast, consuming 24 weeks of HFD containing 20 % olive oil or 20 % butter with 0.1 % cholesterol did not affect significantly this index [66]. The administration of 4-PBA in this study resulted in a rise in insulin levels and a concurrent decrease in glucose levels. Adding the 4-PBA for 30 min to the culture medium of β cells incubated with palmitate was able to improve GSIS and correct the damage caused to these cells [60]. Also, Hong et al. showed that the use of 4-PBA for 3 days (50 mg/kg/day) in male Wistar rats with acute pancreatitis repaired the damaged pancreatic β cells and liver hepatocytes [18,67]. Therefore, in the current study, the 4-PBA probably through inhibiting the phosphorylation of UPR components and as a result inhibiting oxidative and ER stress causes the adverse changes due to HFD consumption to become closer to normal levels and ultimately improves beta cells' function, the plasma levels of insulin and glucose, and the HOMA-β index. The validity of these results was confirmed by an oral glucose tolerance test (OGTT) carried out on the same animal model in our previous study. In this regard, our findings revealed that HFD impaired glucose tolerance by decreasing plasma insulin levels during the OGTT. Nevertheless, the use of 4-PBA resulted in an increase in this parameter, leading to improved glucose tolerance [17].

In this investigation, IDE content in the liver tissue and plasma concentration of insulin significantly decreased in HFD-consuming rats. Contrary to the results of present study, in the hepatic tissue of mice consuming a HFD (58 % kcal fat) for 6 months at the age of 6–8 weeks, the activity and protein content of IDE enzyme was elevated, whereas the mRNA expression of this enzyme was decreased [9]. Moreover, Wei et al. studied the impact of free fatty acids on the expression of IDE and observed that 300 μM palmitic acid enhances the IDE protein level in hepatocytes (Hepa1c7 cells) [9]. There is a suggestion that saturated fatty acids like palmitic acid, which are released from abdominal and mesenteric fat and enter the port system, inhibit the proteolytic and non-proteolytic effects of IDE and reduce insulin removal. In this regard, investigations have revealed that different types of fatty acids quickly reduce the binding of insulin to IDE and the breakdown of insulin by this enzyme within hepatocytes [7]. Furthermore, it has been noted that insulin increases hepatic IDE activity, but this effect is inhibited at high glucose concentrations [7]. It is noteworthy that although the liver is recognized as the primary site for IDE activity and insulin clearance elimination, different organs like kidney and muscle also contribute to insulin clearance through IDE activity. Therefore, it would have been preferable to assess IDE content and activity as well as insulin clearance alterations in these tissues in addition. We recognize this as a limitation of our study and suggest that future studies consider evaluating these parameters in the kidney and muscle [[68], [69], [70], [71]].

Also, IDE has other effects in addition to proteolytic effects. In this regard, preliminary studies in mice showed that global elimination of IDE (IDE-KO-mice) affects the elimination and action of insulin along with insulin release [7]. In these animals' glucose intolerance, increased blood glucose concentration, and insulin signaling disorder, along with upregulation of gluconeogenic genes in liver tissue were observed. These results indicate that IDE performs other functions in relation to glucose homeostasis apart from its involvement in insulin breakdown [72,73]. This research showed that IDE is probably not a rate-limiting regulator of insulin plasma levels *in vivo* [72]. On the other hand, due to the frequent presence of cysteine side chains in the structure of IDE, oxidative and nitrosative processes probably have an influence in the regulation of IDE levels [73]. In this regard, oxidative stress has been proposed as one of the regulators of IDE enzyme activity. IDE enzyme and other enzymes that contain metals in their active site are sensitive to oxidation, which can lead to the loss of the enzyme's catalytic activity. Different pathways have been suggested in relation to oxidative damage to IDE enzyme. It has been revealed that IDE is deactivated by 4-hydroxynonenal (HNE), which is an oxidant agent and a product of lipid oxidative metabolism. NHE, H_2_O_2_ and other oxidant compounds not only suppress the proteolytic activity of IDE but also increase the breakdown of this enzyme by other protein-cleaving enzymes. While, the use of vitamin E supplements, as antioxidant agents that are lipophilic and have a protective role against free radicals, increased the mRNA level of IDE enzyme in the liver of rats, which was linked with improved glucose tolerance and insulin sensitivity [7]. The current study revealed that the reduction in hepatic IDE content was associated with the presence of oxidative and ER stress in the liver tissue. Although the reduction of IDE in the setting of insulin resistance is typically linked to hyperinsulinemia, our findings showed that the decrease in IDE content was coincide with a lower plasma insulin level. Considering IDE's function in insulin secretion, this finding is not entirely unexpected. In this context, Steneberg et al. showed that the elimination of the Ide gene in pancreatic β cells resulted in impaired insulin secretion and reduced granule turnover [74]. Since we did not conduct IDE-specific knockout or inhibitor experiments and we also did not measure IDE activity over time, it is challenging to explain the causal relationship between IDE and oxidative/ER stress as the precise mechanism. These can be regarded as limitations of our study, and they should be addressed in future research.

In this study, 4-PBA injection improved the oxidative stress indices in the liver and increased the hepatic IDE content near the control level. This medication is an aromatic fatty acid authorized by the FDA in the United States to treat urea cycle disorder based on its ammonia scavenging characteristics. At the same time, 4-PBA as a chemical chaperone also inhibits ER stress. In this regard, the results of a study in 8-9-week-old male mice revealed that the use of 4-PBA moderated the indicators of oxidative and ER stress which were increased following exposure of the retina to light stress [75]. In addition, in studies on diabetic rats, the use of 4-PBA improved diabetic retinopathy and nephropathy by reducing ER stress, increasing antioxidant capacity, and reducing lipid peroxidation [76,77]. In fact, there is a communication between oxidative and ER in many physiological and pathophysiological conditions, so that under ER stress conditions, disruption of the control of disulfide bond formation and dissociation can cause an increase in ROS and eventually oxidative stress [78]. Elevated ROS level can also further lead to ER stress by increasing misfolded proteins. On the other hand, the physical and functional association between ER and mitochondria membranes, which modulate the intake of calcium released from the ER into the mitochondria, can activate mitochondrial metabolism and ROS generation. In this process, a self-reinforcing loop of ER and oxidative stress is established [75]. Consumption of a HFD can induce ER stress [79] and oxidative stress [80,81]. In the present study, increased levels of oxidative and ER stress biomarkers were detected in the liver tissue of rats fed a high-fat diet, coinciding with a decrease in IDE content in the liver and plasma insulin level. Given the link between ER and oxidative stress, it is possible that 4-PBA, an inhibitor of ER stress, could potentially restore IDE level in the liver and normalize plasma insulin level by improving the liver's oxidative and ER stress biomarkers.

Moreover, it has been shown that insulin signaling can lead to a moderate but meaningful increase in IDE expression [10]. Given the positive impact of 4-PBA on insulin signaling [15], it is conceivable that 4-PBA enhances liver IDE content by improving insulin signaling.

## 5 Conclusion

In general, our findings showed that chronic intake of a diet high in fat, probably due to dysfunction of pancreatic beta cells, which can be confirmed by decreasing the HOMA-β index, increased plasma glucose concentration and induced hepatic ER and oxidative stress, followed by decreasing the hepatic content of IDE. The findings of this study demonstrated that chronic HFD intake resulted in a decrease in plasma insulin level, which was likely due to pancreatic beta cell dysfunction, as indicated by a reduction in the HOMA-β index. Additionally, the HFD appeared to induce ER and oxidative stress in the liver, along with a decline in liver IDE content. Despite the usual opposite relationship between IDE expression and plasma insulin concentration, the decrease in IDE expression coincided with a decline in plasma insulin level in the current investigation.

## Ethics statement

The current work was done in accordance with the “Ethics Committee of Shahid Beheshti University of Medical Sciences” (IR.SBMU.PHNS.REC.1401.134).

## Funding

Funding for this study was provided by the Neurophysiology Research Center, 10.13039/501100005851Shahid Beheshti University of Medical Sciences, Tehran, Iran [Grant No 43004622].

## CRediT authorship contribution statement

**Fateme Binayi:** Writing – review & editing, Writing – original draft, Visualization, Methodology, Investigation, Formal analysis. **Behnam Saeidi:** Software, Investigation. **Fatemeh Farahani:** Writing – review & editing, Software. **Mina Sadat Izadi:** Investigation. **Farzaneh Eskandari:** Investigation. **Fariba Azarkish:** Investigation. **Mohammad Sahraei:** Investigation. **Rasoul Ghasemi:** Validation, Resources, Data curation. **Fariba Khodagholi:** Validation, Resources, Data curation. **Homeira Zardooz:** Writing – review & editing, Visualization, Supervision, Resources, Project administration, Methodology, Funding acquisition, Data curation, Conceptualization.

## Declaration of competing interest

The authors declare that they have no known competing financial interests or personal relationships that could have appeared to influence the work reported in this paper.
