# Supplementary material for: Sustained feeding of a diet high in fat resulted in a decline in the liver's insulin-degrading enzyme levels in association with the induction of oxidative and endoplasmic reticulum stress in adult male rats: Evaluation of 4-phenylbutyric acid
Source: Heliyon. 2024 Jun 10;10(12):e32804. doi: 10.1016/j.heliyon.2024.e32804 (PMC11226834; doi:10.1016/j.heliyon.2024.e32804)

**Full and non-adjusted figures of Western Blot:**

Original BIP blot:


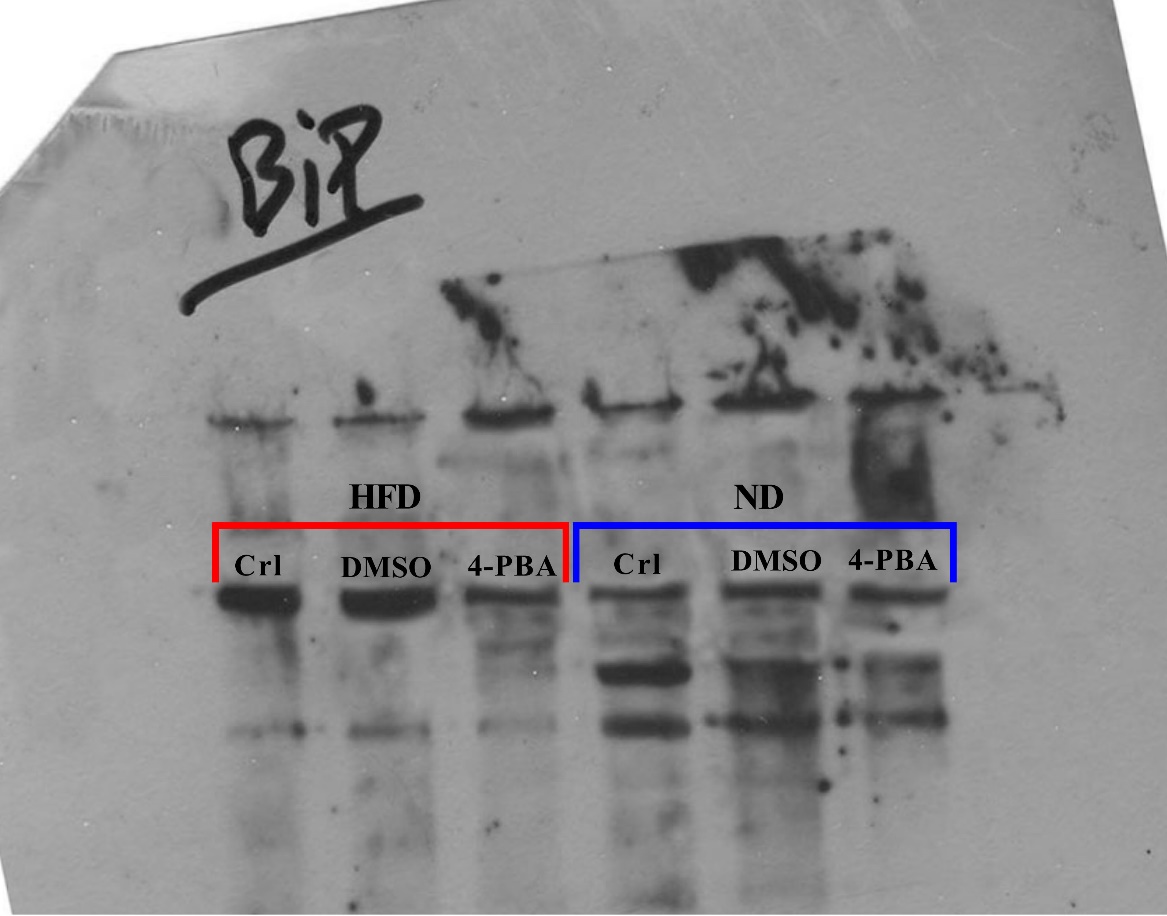


Original CHOP blot:


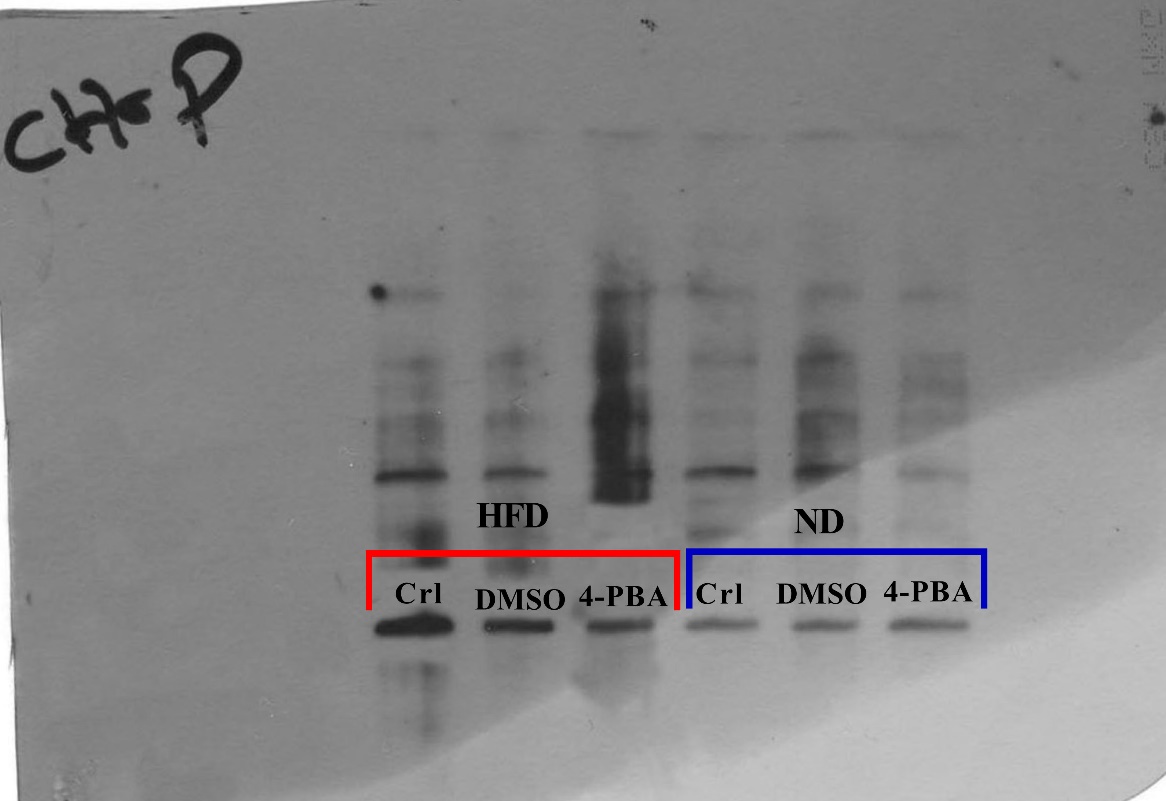


Original β-Actin blot:


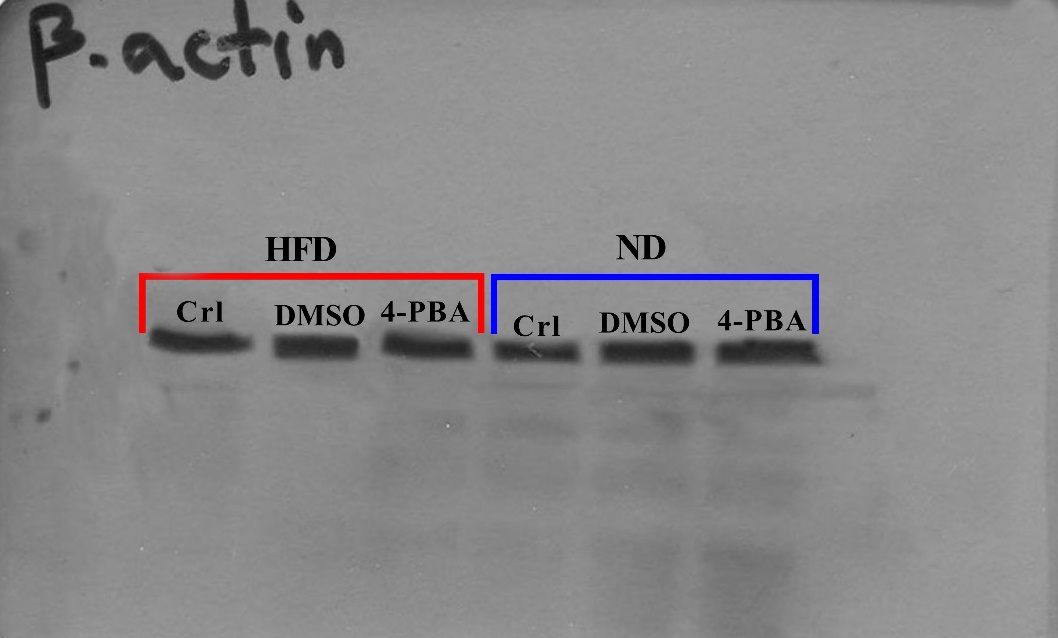

Supplement: Multimedia component 1 [file mmc1.docx]
